# Development and pilot of a decision-aid for patients with bipolar II disorder and their families making decisions about treatment options to prevent relapse

**DOI:** 10.1371/journal.pone.0200490

**Published:** 2018-07-10

**Authors:** Alana Fisher, Louise Sharpe, Josephine Anderson, Vijaya Manicavasagar, Ilona Juraskova

**Affiliations:** 1 The School of Psychology, The University of Sydney, Sydney, New South Wales, Australia; 2 The Centre for Medical and Evidence-based Decision-making (CeMPED), The University of Sydney, Sydney, New South Wales, Australia; 3 The Black Dog Institute, Prince of Wales Hospital, Sydney, New South Wales, Australia; 4 The School of Psychiatry, The University of New South Wales, Sydney, New South Wales, Australia; Brown University, UNITED STATES

## Abstract

**Introduction:**

Treatment decisions in bipolar II disorder (BPII) are finely-balanced and sensitive to patient preferences. This pilot study evaluated a decision-aid booklet (DA) for patients with BPII (and their family) to obtain evidence on its acceptability, feasibility, safety, and usefulness in potential end-users.

**Methods:**

The DA booklet was developed according to International Patient Decision-Aid Standards. Thirty-one patients diagnosed with BPII and their families (*n* = 11), who were currently making or had previously made treatment decisions, participated. Participants read the DA and completed validated and purpose-designed questionnaires. A follow-up semi-structured telephone interview elicited more in-depth DA feedback (*n* = 40).

**Results:**

Patients and family endorsed the DA booklet as: easy-to-use (100% agree), useful in treatment decision-making (100%), presenting balanced (patients = 96.8%, family = 100%), up-to-date (93.5%, 100%) and trustworthy information (93.5%, 100%) that did not provoke anxiety (93.5%, 90.9%). All participants stated that they would recommend the DA to others. Following DA use, all except one participant (97.6%) demonstrated adequate treatment knowledge (> 50% score). Patients reported low decisional conflict (*M* = 18.90/100) following DA use and felt well-prepared to make treatment decisions (*M* = 4.28/5). Most patients (90.3%) indicated uptake of treatments consistent with the best available clinical evidence. Additionally, a large proportion of patients made an informed choice about medication (65.5%) with adjunctive psychological treatment (50.0%), based on adequate knowledge and their treatment values. Interview findings further supported the DA’s acceptability among participants.

**Discussion:**

Pilot findings indicate that patients with BPII and their family consider this DA booklet highly acceptable and useful in making evidence-based treatment decisions that align with their treatment preferences.

## Introduction

A diagnosis of bipolar II disorder (BPII) is commonly accompanied by a need to make complex treatment decisions about mood-stabilising medications and adjunctive psychological therapies, often for lifetime prophylactic use. These decisions are challenging, both from a clinical and a patient perspective. Firstly, there are limited BPII-specific clinical guidelines [1, 2], which reflect limited evidence to support available treatment options in individuals with BPII [3, 4]. Next, there are a number of viable medical and adjunctive psychological treatment options available with varying benefit/cost profiles. Some medication options can have significant potential side-effects, for example, cognitive dulling and weight gain [5, 6], which some patients may perceive as outweighing any immediate therapeutic benefits. Thus, these “preference-sensitive” treatment decisions need to incorporate the best available clinical evidence, clinician judgement, and patient preferences [7].

To date, no known resources have been developed to facilitate more informed and active patient (and family) involvement in BPII treatment decision-making. Patient decision-aids (DAs) are evidence-based interventions for potentially improving shared treatment decision-making (SDM) in BPII. DAs are designed to help patients make specific and deliberative healthcare choices, by weighing up the pros (‘benefits’) and cons (‘costs’) of all available options whilst considering their personal values/preferences. Emerging evidence from randomised controlled trials (RCT) supports the effectiveness of six known mental health treatment DAs, for unipolar depression [8–10], post-traumatic stress disorder [11, 12], and schizophrenia [13]. In light of these promising findings, and in the absence of any BPII-specific DAs, this pilot study aimed to:

1. obtain preliminary evidence on the acceptability, safety, feasibility and potential usefulness of a newly-developed DA booklet for patients with BPII and their family making decisions about prophylactic treatment (for relapse prevention); and
2. establish the feasibility, relevance and acceptability of the procedures and measures used, to inform the design of a RCT evaluation of the DA.

## Methods

### Participants

#### Patients

Adults with a clinical diagnosis of bipolar II disorder (BPII) who were currently making or had previously made decisions about their treatment (medical or non-medical) were eligible to participate.

#### Family members

Adults whose family member had: i) an adult BPII diagnosis (18+ years), and who had ii) attended at least one consultation involving treatment decision-making, and/or had iii) experience helping their family member make treatment decisions outside consultations were also invited to participate. Patient participation was *not* a pre-requisite for family member participation.

Exclusion criteria for both samples were: i) insufficient English proficiency; ii) inability to provide informed consent; ii) (comorbid) substance abuse disorder; iv) other major psychiatry/neurological disorder or cognitive impairment.

Ethics approval was obtained from the University of Sydney (USYD) Human Research Committee and the Black Dog Institute (BDI) Research Advisory Board; the study was carried out according to the principles outlined in the Declaration of Helsinki [14].

Participants were recruited through the following pathways: A. patient referrals to BDI (with family members identified through patients) an outpatient clinical service specialising in the assessment and treatment of mood disorders; B. patient/family attendees at the BDI’s BPII support groups; C. purposively-sampled participants from previous research [15] who had agreed to be contacted regarding future research participation; D. members of Australia-based online community forums/social-media platforms for people affected by mood disorders (patients and family) (*BeyondBlue*, *SANE* and *Livin’*).

The use of multiple recruitment pathways ensured a mix of participants who were *actively* considering treatment options—the DA target population (i.e. pathway A); or who had *already* made a BPII treatment decision (i.e. pathways B-D).

For patients recruited through pathways A-C, BPII diagnosis was based on a “consensus diagnostic decision” between at least two assessing psychiatrists with expertise in mood and bipolar disorders [16]. To establish BPII diagnosis, all patients were clinically assessed by an intake psychiatrist who made a lifetime clinical diagnosis of BPII applying clinician-judged criteria. These criteria took into account DSM-5 symptom criteria [17] but did not impose the minimum duration criterion for hypomania (4 days). This criterion is largely arbitrary and not of clinical significance [16, 18]. Approximately a third of these patients were also assessed by a second independent psychiatrist. Prior to clinical assessment, patients also completed the 27-item Mood Swings Questionnaire [19], which has sensitivities and specificities of 70–82% and 78–98% in tertiary patient referral samples [20, 21]. For patients recruited through pathway D, BPII diagnosis was based on self-report. We required, however, that these patients had been diagnosed with BPII by a mental health specialist (i.e., psychiatrist) as opposed to general physician (GP) (i.e., primary care provider).

### Procedure

For the patient referral sample (pathway A) a clinic research assistant introduced the study to eligible patients following their clinical assessment, and gave the contact details of interested patients to the study coordinator at USYD (AF). Purposely-sampled participants (pathway C) were contacted directly by AF via their provided contact details to introduce the study and ascertain their interest in participating. All other participants responded to an expression-of-interest flyer, which was disseminated via the support group meetings and online forums (pathways B and D, respectively).

AF telephoned interested participants to explain the nature and purpose of the study and obtain verbal consent to post/email a study pack containing: an information sheet and consent form, a copy of the DA booklet and a study questionnaire. Upon receiving the completed questionnaires and written consent form, a one-off telephone interview was arranged.

### Materials and measures

#### The BPII decision-aid (DA)

The BPII DA booklet was informed by the International Patient Decision-Aid Standards (IPDAS) [22] and the Ottawa Decision-Support Framework [23]. Content, formatting and design were based on: a systematic review [24], the best available evidence (e.g., clinical guidelines [1, 2, 25], meta-analyses [26, 27] and well-designed, placebo-controlled RCTs [28–34]); in-depth qualitative interviews with patients (*n* = 28), family (*n* = 13), and clinicians (*n* = 20) [15, 35, 36]; and iterative review by an expert working party. The BPII DA was a 100 page A5 booklet, with information divided into three main sections (via dividing tabs): Medication Options, Psychological Options, and Making Decisions. Throughout, the DA provides evidence-based, lay information using text and graphics on the known efficacy and benefits/costs of the current first-line medications (e.g., lithium, lamotrigine, quetiapine) [1, 2] and Level-1 evidence-supported psychological treatments (e.g., individual cognitive behavioural therapy [CBT], group psycho-education) [25] for relapse prevention in BPII specifically. Values clarification exercises (VCE’s) help patients/family consider their preferences and deliberate on the benefits/costs of the different treatment options. Other (i.e., second-line and/or adjunctive) medications and psychological treatment options were excluded due to limited data supporting their efficacy specifically for patients with BPII. Including these other options would be superfluous to the main purpose of a DA, which is to support patients facing ‘preference-sensitive’ decisions. That is, deciding between treatment options that are supported by *similar* evidence, and thus clinical uncertainty remains with regards to which option is superior (i.e., equipoise) [37].

The DA’s readability levels were not assessed, as readability may not be an appropriate index of comprehensibility when patient information materials contain multisyllabic medical terminology [38]. This terminology were necessary to include and were defined in simple, descriptive terms in the DA’s glossary. As a more appropriate alternative to assessing readability levels, the DA was professionally copy-edited for low health literacy levels. In addition, health literacy review using the Patient Education Materials Assessment Tool (PEMAT; Shoemaker [39]) yielded “understandability” scores of 76%, placing the DA in the “superior” range for easy to understand and use patient education materials [40].

The DA is designed for patients/family to use before and/or after clinical consultations in which treatment options for relapse prevention/maintaining mood stability are discussed. Thus, the DA is *not* intended to replace treatment discussions with their managing clinician, but rather support and prepare patients to have these discussions. See S1 Appendix for a full summary of the DA booklet content.

#### Interview guide

The purpose-designed, semi-structured interview guide (see S2 Appendix) was informed by the Ottawa Acceptability measure [41]. Open-ended questions elicited feedback on the DA’s acceptability and suggested improvements.

#### Measures

Purpose-designed and validated measures evaluated the DA’s acceptability and potential usefulness in terms of key decision quality constructs [42]. Asterixed measures (*) were completed by patients only.

*Participant DA feedback* was assessed using an adapted measure from previous acceptability studies of mental health decision-support [43]. Participants indicated their agreement on the DA’s *perceived ease of use* (8 items), *perceived usefulness* (9 items), *attitudes towards using* (3 items), and *perceived trustworthiness/bias* (4 items). Four agreement categories were collapsed into agree (agree/somewhat agree) and disagree (disagree/somewhat disagree).

#### Measures of decision-making quality

Perceived difficulties with decision-making* were assessed using the 16-item validated Decisional Conflict Scale (DCS; *α*’s = 0.78–0.92) [44]. Five subscales measured patients’ feelings of being: i) uncertain about the treatment options, ii) uninformed, iii) unsupported, iv) unclear about values in decision-making, and v) unable to make an effective decision (scores 0–100). A total score (0–100) indicated overall decision-making difficulties. Lower scores denoted less decision-making difficulty.

*Objective knowledge* of treatment options and outcomes employed a competency-based approach [45], whereby 14 forced-choice items assessed conceptual/gist (9 items yielding possible total scores 0–18; “*true*”, “*false*”, “*don’t know*”) and numerical/verbatim (5 items yielding possible total scores 0–20; A-E responses) knowledge of information contained in the DA. Assessed domains were based on current NHMRC guidelines for medical practitioners on giving information to patients for informed consent purposes [46]. Based on Smith et al. [45], responses were scored according to an *a priori* marking scheme, with the threshold for adequate knowledge for informed choice (*yes/no*) set at > 50% of total possible score (i.e., score of 20 or more out of 38) (S3 Appendix).

*Subjective/perceived knowledge* of treatment options and outcomes was assessed via a 15-item purpose-designed measure, whereby participants indicated how well they had understood (1 = *didn’t understand at all* to 5 = *understood very well*) information contained in the DA. Again assessed knowledge domains were based on current NHMRC guidelines [46].

*Informed*, values-based choice* was determined via a composite measure of objective knowledge (see above), attitudes, and treatment choice. Attitudes towards medication and psychological options were assessed using two Likert-type scales, which each contained four items. Each item was anchored by opposing positive/negative adjectives (e.g., 1 = *Beneficial*, 7 = *Harmful*) [47]. Patients also indicated their (hypothetical) treatment choice after reading the DA (e.g., medication/s with/out adjunctive psychological treatment versus no medication/treatment). Patients were defined as making an informed choice if they had adequate objective knowledge (i.e., > 50%) [45] and made a treatment choice that was consistent with their values (e.g., *positive* attitudes towards medication/s *plus* indicating intentions to take up medication/s) [47]. A median split categorised patients with positive attitudes (≥median) or negative attitudes (<median) [47].

*Preparation for decision-making* scale* assessed via 10 items patient perceptions of the DA’s usefulness in preparing them to make treatment decisions (*α*’s = 0.92–0.96) [48]. Each item was rated from *not at all = 1* to *a great deal = 5* yielding a mean possible score of 1–5.

#### Measures of sample characteristics

*Anxiety levels* were assessed using the 6-item short form of the State Trait Anxiety Inventory (STAI-Y-SF) [49].

Symptom severity/mood state* within the past 24 hours was self-reported using the 17-item Internal States Scale (ISS) [50]. Each item was rated from 0 (*rarely*/*not at all*) to 100 (*very much so*/*most of the time*). The combination of total scores on the Activation (<155 or ≥155) and Wellbeing (<125 or ≥125) subscales indicated the patient’s current mood state.

*Stage of decision-making* scale categorised patient’s (lack of) readiness to engage in decision-making, from not thinking about treatment choices (item 1) to actively deliberating on options (item 3) to having already made a treatment decision (item 5) [51].

*Preferred and experienced levels of patient involvement in decision-making* were assessed via two administrations of the single-item adapted Control Preferences Scale [52, 53]. Concordance/discordance was indexed via (dis)agreement between the two ratings [54, 55].

*Information preferences* were assessed using an adapted version of the Cassileth Information Styles questionnaire [56]. Items elicited preferences regarding the amount (1–5) and type (*enough to care for self*; *only good*; *all information*, *good and bad*) of medical information.

*Health literacy* was measured via the Single Item Literacy Screener (SILS) [57]: “*How often do you need to have someone help you read instructions*, *pamphlets*, *or other written material from your doctor or pharmacy*?”. (*never* = 1 to *always* = 5). Scores of >2 reflect some difficulties understanding written health materials. To control for mood symptoms as a potential confound, the item was reworded for patients to include: *“When not experiencing symptoms of depression or hypomania…”*

*Demographic*, *clinical and family involvement information* was obtained via a purpose-designed self-report questionnaire.

#### Data analyses

Descriptive and frequency analysis of the quantitative questionnaire data used IBM SPSS 23. Qualitative analyses of participants’ interview responses used a thematic approach [58] to inform the relevant quantitative findings.

## Results

### Sample demographics

Of the 49 patients and 20 family members who agreed to participate, 30 patients and 10 family members completed both the questionnaire and follow-up interview lasting approximately 30 minutes on average (response rates: 61.2% and 50%, respectively). An additional one patient and one family member completed the questionnaire only. Due to the way in which participants were approached for this study (e.g., patient referrals from a private clinic), limited information is available for those patients and family who agreed to participate but did not go on to complete study procedures. Of those participants who were able to be contacted, reasons for non-participation included: lack of interest and time/other competing commitments (n = 2), significant change in personal circumstances (moving overseas, undergoing divorce, n = 2), not receiving the study package in the post (n = 2), and hospitalisation for mood symptoms (n = 1).

Sample demographics are summarised in Table 1. Patients were aged on average 36.67 years, (*SD* = 12.63), and family on average 46.64 years (*SD* = 15.87). Both samples comprised mostly women (77.4% patients, 81.8% family), the majority were Australian-born (80.6%, 72.7%) with university level education (58.1%, 63.7%) and engaged in part-time/full-time work (70.9%, 72.8%).

**Table 1:** Demographic characteristics of patient (*n* = 31) and family (*n* = 11) samples.

	Patients	Family
	***M* (*SD*)**	***M* (*SD*)**
**Age**	36.87 (12.63)	46.64 (15.87)
	**n (%)**	**n (%)**
**Gender**		
Female	24 (77.4)	9 (81.8)
**Relationship to patient**		
Parent	--	5 (45.5)
Spouse/partner	--	3 (27.3)
Sibling	--	2 (18.2)
Child	--	1 (9.1)
**Highest qualification**		
Year 12/ HSC or below	7 (22.6)	2 (18.2)
TAFE certificate/diploma	6 (19.4)	2 (18.2)
University degree	14 (45.2)	4 (36.4)
Postgraduate degree	4 (12.9)	3 (27.3)
**Current employment**		
Working full-time	13 (41.9)	4 (36.4)
Working part-time	9 (29.0)	4 (36.4)
Studying	3 (9.7)	--
Not employed/retired/home-duties	5 (16.1)	3 (27.3)
Other (e.g., part-time work & study)	2 (6.5)	--
**Country of birth**		
Australia	25 (80.6)	8 (72.7)
Other (e.g., UK, Japan)	6 (19.4)	3 (27.3)
**Language spoken at home**		
English	30 (96.8)	11 (100)
Other (Turkish)	1 (3.2)	--
**Current relationship status**		
Married/living with partner	17 (54.8)	10 (90.9)
Single/dating	10 (32.3)	--
Separated or divorced	4 (12.9)	1 (9.1)
**Current living arrangement**		
With partner (with/out children)	16 (51.6)	9 (81.8)
By yourself/independently	5 (16.1)	2 (18.2)
With other family members	5 (16.1)	--
With non-family members	5 (16.1)	1 (9.1)
**Patient/family participant pairs**	--	3 (27.3)
Yes	--	3 (27.3)

#### Clinical and family involvement characteristics

As seen in Table 2, an equal number of patients reported having a recent (< 12 months, 41.9%) or longer-standing BPII diagnosis (1–5 years ago, 41.9%). Meanwhile, over half of family participants had a family member with a longer-standing BPII diagnosis (54.5%). Both patients and family participants indicated slightly elevated anxiety at the time of the study (~one *SD* above age-matched community norms, *M* = 46.56, 44.55, respectively).

**Table 2:** Clinical characteristics of patient (*n* = 31) and family (*n* = 11) samples.

	Patients	Family
	***M* (*SD****)*	***M* (*SD*)**
**Age diagnosed with BPII**	34.16 (11.96)	32.64 (12.96)
**State anxiety (20–80)**	46.56 (13.23)	44.55 (15.72)
	***n* (%)**	***n* (%)**
**Time since BPII diagnosis**		
<1 month	5 (16.1)	--
1–12 months	8 (25.8)	4 (36.4)
1–5 years	13 (41.9)	6 (54.5)
5 + years	5 (16.1)	1 (9.1)
**BPII episodes—(perceived) frequency**		
More than once per month	10 (32.3)	2 (18.2)
4 or more times per year	11 (35.5)	3 (27.3)
2–3 times per year	5 (16.1)	5 (45.5)
About once per year	4 (12.9)	--
Less than once per year	1 (3.2)	1 (9.1)
**BPII episodes—(perceived) type**		
Mainly depressive episodes	15 (48.4)	3 (27.3)
Equal depression/hypomania	11 (35.5)	6 (54.5)
Mainly hypomanic episodes	4 (12.9)	1 (9.1)
Mainly euthymic/subsyndromal	1 (3.2)	1 (9.1)
**Current mood state (ISS)**		
Hypomania	13 (41.9)	--
Euthymia	7 (22.6)	--
Depression	6 (19.4)	--
Mixed state	5 (16.1)	--
**Current medication/s**		
Mood stabiliser only (incl. anticonvulsants)	14 (45.2)	7 (63.6)
Atypical antipsychotic	--	1 (9.1)
Antidepressant	2 (6.5)	--
Mood stabiliser plus atypical antipsychotic	2 (6.5)	--
Mood stabiliser plus antidepressant	4 (12.9)	--
All three types	4 (12.9)	1 (9.1)
No medication	5 (16.1)	2 (18.2)
**Current psychological treatment**		
Yes (e.g., CBT, counselling)	19 (61.3)	4 (36.4)
**Current goal of BPII treatment**		
Prevent recurrence/relapse	24 (77.4)	9 (81.8)
Treat current depression	3 (9.7)	--
Treat current hypomania	--	1 (9.1)
Other (e.g., combination of above)	4 (12.9)	--
Don’t know	--	1 (9.1)
**Family attended consultation/s**		
Yes	22 (71)	9 (81.8)
**Usual attendance in consultation/s**		
Usually patient alone	24 (77.4)	9 (81.8)
Attends accompanied	3 (9.7)	1 (9.1)
Sometimes alone or accompanied	4 (12.9)	1 (9.1)

Patients and family reported that they/their family member experienced mainly depressive or equal depressive/hypomanic episodes (83.9, 81.8%, respectively). Almost half of patients (45.2%) and two-thirds of family participants (63.9%) reported that they/their family member was currently taking a mood-stabiliser medication. Around two-thirds of patients (61.3%) and a third of family (36.4%) reported that they/their family member was undertaking psychological treatment. Most patients and family nominated relapse prevention as their/their family member’s current treatment goal (77.4%, 81.8%, respectively).

Most patients and family participants indicated that family had attended at least one consultation regarding BPII treatment (71%, 81.8%, respectively), however, patients usually attended consultations alone/unaccompanied (77.4%, 81.8%).

### Pre-existing decision-making characteristics

#### Information preferences and decision-making stage

Both patients and family preferred to receive a large amount of information (*M* = 4.82, 4.91/5, respectively) and most wanted “as much information as possible, good or bad” (87.1, 90.9%, respectively) (Table 3). In terms of decision-making stage, 77.4% of patients and 63.6% of family indicated that they/their family member were either currently considering treatment options, or had already made a treatment decision but were willing to reconsider these options. No participants indicated health literacy-related difficulties (scores < 2).

**Table 3:** Pre-existing decision-making characteristics of patient (*n* = 31) and family (*n* = 11) samples.

	Patients		Family	
	***M* (*SD*)**		***M* (*SD*)**	
**Information preferences—*amount* (/5)**	4.82 (0.42)		4.91 (0.30)	
	***n* (%)**		***n* (%)**	
**Information preferences—*type***				
As much information as possible, good or bad	27 (87.1)		10 (90.9)	
Only information to take care of myself/my family	4 (12.9)		1 (9.1)	
**Involvement in decision-making (dyadic)**	***Pref***	***Exp***	***Pref***	***Exp***
Patient-led with/out clinician^b^	12 (38.7)	14 (45.2)	8 (72.7)	9 (81.8)
Shared/collaborative	12 (38.7)	9 (29.0)	3 (27.3)	1 (9.1)
Clinician-led with/out patient^c^	7 (22.6)	8 (25.9)	--	1 (9.1)
**Involvement in decision-making (triadic)**	***Pref***	***Exp***	***Pref***	***Exp***
Patient-led with/out clinician/family^b^	22 (71)	22 (71)	11 (100)	10 (90.9)
Shared/collaborative	2 (6.5)	2 (6.5)	--	--
Clinician-led with/out patient/family^c^	7 (22.6)	6 (19.4)	--	--
No family involvement	--	1 (3.2)	--	--
**Experienced preferred level of patient involvement (dyadic)**				
Yes	19 (61.3)		4 (36.4)	
**Experienced preferred level of patient involvement (triadic)**				
Yes	17 (54.8)		8 (72.7)	
**Patient decision-making stage**				
Not begun to think about choices but interested	1 (3.2)		--	
Considering options now	9 (29.0)		--	
Already made a decision, willing to reconsider	15 (48.4)		7 (63.6)	
Already made a decision, unlikely to change mind	6 (19.4)		4 (36.4)	
**Read the DA**				
Just briefly	1 (3.2)		1 (9.1)	
Just parts relevant to me	3 (9.1)		2 (18.2)	
Quite thoroughly	8 (25.8)		4 (36.4)	
From cover to cover	19 (61.3)		4 (36.4)	

Notes:

^a^ Participants indicated perceived/experienced levels of clinician-patient(-family) involvement in the most recent consultation involving BPII treatment decision/s. This consultation may have occurred prior to/after exposure to the DA.

^b^ Combines “fully patient-led decision-making” and “patient-led decision-making with clinician (and family) input”.

^c^ Combines “fully clinician-led decision-making” and “clinician-led decision-making with patient (and family) input”.

#### Preferred and experienced involvement in decision-making

As with information, patients and family also indicated strong preferences for patient involvement (Table 2). Overall, patients and family mostly preferred and experienced either patient-led or shared decision-making in consultations involving BPII treatment decision/s. A smaller proportion of patients compared to family preferred and experienced patient-led decision-making. Further, patients more often than family preferred and experienced shared or clinician-led decision-making in consultations (Table 2).

Regarding concordance, 61.3% of patients (*n* = 19) and 36.4% of family (*n* = 4) experienced their preferred level of patient involvement in the most recent *dyadic (clinician-patient)* consultation involving BPII treatment decision/s (Table 2). By contrast, 54.8% of patients (*n* = 17) and 72.7% of family (*n* = 8) experienced their preferred patient level of involvement in the most recent *triadic* (*clinician-patient-family*) consultation involving BPII treatment decision/s (Table 2).

#### Read the DA

All participants reported reading the DA and most also indicated good engagement; 87.1% of patients and 72.8% of family participants read the DA “quite thoroughly” or “cover to cover” (Table 2). Participants were not asked how long it took them to read through the DA, however, participants were expected (and encouraged) to review the DA over a number of sittings (as opposed to a single sitting). This said, participants who volunteered this information during interviews noted that reading through the DA took approximately 40–45 minutes.

### Decision-making quality characteristics

#### Uptake of effective treatment option

After reading the DA, almost all patients (90.3%) indicated that they would take up an effective treatment option: mostly a medication option (48%) or combination of medication/s plus an adjunctive psychological treatment (41.9%, Table 4). Remaining patients (*n* = 3, 9.7%) indicated that they were unsure or chose to delay treatment uptake.

**Table 4:** Decision-making quality characteristics of patient (*n* = 31) and family (*n* = 11) samples.

	Patient	Family
	***n* (%)**	***n* (%)**
**Uptake of effective treatment options** (as per DA)^a^		
Medication/s only	15 (48.4)	--
- Lithium	1 (3.2)	--
- Lamotrigine	8 (25.8)	--
- Quetiapine	1 (3.2)	--
- Combination of above medications	2 (6.5)	--
Medication/s plus adjunctive psychological treatment	13 (41.9)	--
No treatment uptake/ unsure	3 (9.7)	--
	***M* (*SD*)**	***M* (*SD*)**
**Decisional conflict** (/100)^a^		
Total	18.90 (13.90)	--
Uncertainty	30.11 (24.97)	--
Informed	13.98 (14.65)	--
Values	8.87 (12.16)	--
Support	15.32 (14.45)	--
Effective decision	24.40 (19.25)	--
**Preparation for decision-making** (/5)	4.28 (0.61)	--
**Subjective understanding of treatment options** (/5)	4.45 (0.57)	4.36 (0.50)
**Objective knowledge of treatment options**		
Total score (/38)	32.04 (4.43)	34.41 (3.81)
Conceptual/gist knowledge (/18)	15.16 (2.72)	16.73 (1.85)
Numerical/verbatim knowledge (/20)	16.87 (2.88)	16.32 (5.18)
	***n* (%)**	***n* (%)**
**Adequate level knowledge of treatment options** (Total score > 50%, see S3 Appendix for scoring)*	30 (96.8)	11 (100)
**Attitudes medication options** (as per DA)*		
Positive (at or above median)	20 (64.5)	8 (72.7)
Negative (below median)	9 (29)	3 (27.3)
**Attitudes adjunctive psychological options** (as per DA)*		
Positive (at or above median)	21 (67.7)	7 (63.6)
Negative (below median)	9 (29)	4 (36.4)
**Informed treatment choice (yes)**^a^		
Medication/s uptake	19 (65.5)	--
Medication/s plus adjunctive psychological treatment uptake	15 (50.0)	--

^a^ = based on hypothetical choice/uptake after reading the DA.

* Note remaining percentage = missing data.

#### Decision-making difficulties and preparation

With regards to their hypothetical treatment choice, patients indicated low levels of decisional conflict on their total score (*M* = 18.90/100) and on each of the subscales (*M* = 8.87–30.11/100) (Table 4), on average. Only the uncertainty subscale had average scores over 25 (30.11/100), indicating that some patients felt unsure/unclear about which option was best for them. On average, patients also indicated that the DA prepared them well to make treatment decisions (*M* = 4.28/5).

#### Knowledge and understanding of treatment options

Patients and family reported good subjective understanding of the DA treatment options and outcomes (*M* = 4.45, 4.36/5, respectively, Table 4). Objective knowledge was similarly high; patients and family were highly knowledgeable in terms of average total (*M* = 32.04, 34.41/38, respectively), conceptual and numerical knowledge (Table 4). Accordingly, all but one patient demonstrated adequate knowledge (i.e., > 50% of possible total score, S3 Appendix). Additional post-hoc analyses were conducted on adequate knowledge about treatment options and outcomes. Using a cut-off score of >75% instead of >50%, these analyses revealed that, even with the more stringent cut-off score, the large majority of both patient (*n* = 24, 77.4%) and family (*n* = 9, 81.8%) participants still demonstrated adequate knowledge of treatment options and outcomes.

#### Attitudes towards treatment options and informed treatment choice

The majority of patients and family expressed positive attitudes towards the presented medications (64.5%, 72.7%, respectively) and adjunctive psychological treatments (67.7%, 63.6%, respectively) for BPII relapse prevention (Table 4).

Based on congruence between patient’s knowledge and treatment attitudes, 65.5% made an informed choice about medication uptake and 50.0% made an informed choice about taking-up adjunctive psychological treatment. All remaining patients made a treatment choice that was based on adequate knowledge (except *n* = 1) but was incongruent with their treatment attitudes (e.g., negative attitudes towards medication, yet decided to take-up medication).

### Participant feedback on the DA

Participant feedback on the booklet was highly positive across all acceptability domains (Table 5). The qualitative interview data reflected these mostly positive attitudes. Differences between patients and family or those participants with a recent (< 12 months) versus longer-standing (1 year +) diagnosis are noted below. These differences were minimal overall, however. For illustrative participant quotes, see Table 6.

**Table 5:** Quantitative participant feedback on the decision-aid (DA) in the patient (*n* = 31) and family (*n* = 11) samples.

	Patients		Family	
	Agree/ Somewhat Agree *n* (%)	Disagree/ Somewhat Disagree *n* (%)	Agree/ Somewhat Agree *n* (%)	Disagree/ Somewhat Disagree *n* (%)
**Perceived ease of use of DA**				
Font easy-to-read	31 (100)	--	11 (100)	--
Easy-to-use	31 (100)	--	11 (100)	--
Clearly organised information	30 (96.8)	1 (3.2)	11 (100)	--
Design appealing	31 (100)	--	11 (100)	--
Easy-to-understand information	31 (100)	--	11 (100)	--
Colours pleasant	31 (100)	--	11 (100)	--
Pictures relevant	31 (100)	--	11 (100)	--
Important information easy-to-find	31 (100)	--	11 (100)	--
**Perceived usefulness of DA**				
Content interesting	31 (100)	--	11 (100)	--
Useful in making a treatment decision	31 (100)	--	11 (100)	--
Right amount of information included	31 (100)	--	10 (90.9)	1 (9.1)
Information I needed included	31 (100)	--	10 (90.9)	1 (9.1)
Helped with my concerns	30 (96.8)	1 (3.2)	10 (90.9)	1 (9.1)
Found links to information and other resources	28 (90.3)	3 (9.7)	11 (100)	--
Learnt something new	29 (93.5)	2 (6.5)	11 (100)	--
Made it easier to discuss treatment options with family	26 (83.9)	4 (12.8)	10 (90.9)	1 (9.1)
Made it easier to discuss treatment options with (my) clinician*	28 (90.3)	3 (9.7)	10 (90.9)	--
**Attitudes towards using DA**				
Would recommend to others in my situation	31 (100)	--	11 (100)	--
Would go back and re-read sections	30 (96.8)	1 (3.2)	11 (100)	--
Information did *not* make me anxious (safety check)	29 (93.5)	2 (6.5)	10 (90.9)	1 (9.1)
**Perceived trustworthiness and balance of information in DA**				
Information trustworthy*	29 (93.5)	1 (3.2)	11 (100)	--
Information up-to-date*	29 (93.5)	1 (3.2)	11 (100)	--
Equal emphasis placed on each of the medication options	30 (96.8)	1 (3.2)	11 (100)	--
Equal emphasis placed on each of the adjunctive psychological options	31 (100)	--	11 (100)	--

*Remaining percentage = missing data.

**Table 6:** Illustrative participant quotes on DA acceptability feedback.

Acceptability domain	Illustrative participant quotes
Perceived ease of use	*“…I liked the tabs*, *{made the DA…] easy to navigate… [I] liked how it [the DA] is set out*, *very user friendly*, *clear and well explained and easy to read…”* (Patient ID143, female 24 yrs, dx < 1 month) *“…[I liked the use of…] calming and neutral colours*. *Subsections useful in helping to locate info*. *Design is good and the text was broken up into small sections; this made a good balance between the text and the images*, *diagrams…”* (Patient ID107, male 28 yrs, dx > 5 yrs)
Perceived usefulness	*“…[The DA is…] the most solid thing I’ve got in terms of knowing the options and not just relying on the psychiatrist and the psychologist and their recommendations*. *You can tailor the options to you and you can decide the side effects that are worth while and give more control*.*”* (Family ID210, wife of 40 yrs male patient dx 2 yrs). *“…[The DA was…] really helpful*. *The information was in-depth and gave you a good clear understanding of the options*. *[It’s a*..*] useful tool… when you’re first diagnosed you don’t know where to start and are reliant on medical professionals*.*”* (Patient ID123, female 50 yrs, dx 4 yrs)
Attitudes towards using	*“… Seeing some of the negative*, *side effects can be daunting but I’m someone who likes to know everything…”* (Patient ID120, female 32 yrs, dx < 1 month) *“… Probably the fact that it [the DA] talks about family involvement and helping with the decision-making […made me anxious]*. *We’ve not really been involved*. *[But]*… *after reading that I went to see my son’s psychiatrist to see how I can help him manage better*.*”* (Family ID219, mother of 28 yrs, male patient dx 3 yrs)
Perceived trustworthiness and balance	*“…the information [in the DA was] straight-up*, *not biased at all”* (Patient ID115, female 23 yrs, dx 2 yrs) *“…[the DA] just gave the evidence as it is…”* (Patient ID118, male 46 yrs, dx > 5 yrs) *“…[the DA’s balanced view] helped with making one’s own informed decision…”* (Patient ID120, female 32 yrs, dx < 1 month)

#### Perceived ease of use

All participants except one patient agreed that the DA was easy-to-use, and contained information that was easy-to-understand and clearly-organised (Table 5). Qualitative feedback echoed this, with most participants commenting that the DA was well-laid out and provided *“plain” “straightforward”* information, with balanced use of text and graphics (Table 6, IDs 143, 107). About half of participants (n = 17), in particular patients (n = 16), felt that it would be helpful to have a clinician go through the DA to introduce medications and highlight the different DA sections.

#### Perceived utility

All patients and all except one family member agreed that overall, the DA was useful for making a treatment decision-making (Table 5). Despite this, several participants, especially those with a longer-standing diagnosis, indicated that the information in the DA did not specifically: help them with their concerns, provide them other resources, teach them something new, and/or make it easier to discuss treatment options. Participants commented that the DA was a *“good starting point*” and especially useful for those with a recent BPII diagnosis because it clearly summarised the main available options in terms of their pros (e.g., efficacy) and cons (e.g., side-effects). Participants reported that the visual aids (e.g., colour-coded summary tables, 100-person dot diagrams) enhanced the DA’s usefulness, because they permitted cross-comparisons between the different treatment options in a structured and guided way. Several participants commented that access to comprehensive and specific benefit/risk information helped them to feel more informed, in control, and “*active consumers”* (Table 6, IDs 210, 123).

The usefulness of DA section on family member involvement in treatment decision-making revealed somewhat mixed views. Some patients and family—who had a recent diagnosis or were yet to involve family/be involved—found this section increased their awareness of the practical ways of involving family and/or served as an impetus to involve family. Ten patients and two family participants explained that this section had limited relevance to them as family were not involved, or they had already involved family.

#### Attitudes towards using the DA

All participants agreed that they would recommend the DA to others in their situation (Table 5). Two patients and one family member indicated that reading the DA made them feel anxious. During interviews, these participants attributed their anxiety to reading about the more “serious” side-effects and incomplete efficacy of medications at preventing relapse, yet they endorsed this information as necessary and important (Table 6, IDs 120, 219). Contrastingly, a few participants noted that reading the DA reduced their anxiety because the information provided them with reassurance and a sense of “control”.

#### Perceived trustworthiness and balance

Participants agreed that the DA provided a trustworthy, unbiased presentation of the treatment options (Table 5). This positive feedback was reiterated in interviews (Table 6, IDs 115, 118, 120).

Nine participants, mainly those with a longer-standing diagnosis, suggested that the DA includes a clearer rationale for selecting lithium, lamotrigine and quetiapine as provided medication options, and emphasise that other options are available; and explain that patients may need to supplement these medications or try other medications.

Of note, most patients (*n* = 24) and family (*n* = 7) felt that the inclusion of patient/family quotes was helpful in giving positive but realistic expectations of treatment outcomes. The quotes were endorsed as a valuable *“person-based”* supplement to the *"clinical"* and *"statistical"* type information presented.

#### Other qualitative findings—Suggested changes and additions

Half of patients (*n* = 15) and most family members (*n* = 6) did not suggest including any additional DA content. Suggested additions included: more information on the evidence base relating to complementary therapies (e.g., exercise, mindfulness); clarification on other commonly-prescribed medications for bipolar (e.g., sodium valproate); and the fact that finding the ‘right’ medication offering the most therapeutic benefit and fewest side-effects takes time.

## Discussion

This paper reports on the development and pilot of the first BPII-specific decision-aid (DA) to assist patients and their families to make decisions about treatment options to prevent relapse. Quantitative and qualitative feedback provided evidence of the DA’s acceptability in terms of its perceived ease of use, usefulness, trustworthiness and balance, and attitudes towards using the booklet. Evidence of safety using the DA was derived from participant ratings of whether the DA information provoked anxiety/stress, along with state anxiety levels. Feasibility evidence was derived from the pilot process itself, and identifying any recruitment or procedure-related challenges. Evidence of the DA’s potential usefulness in improving BPII treatment decision-making was assessed via numerous measures of decision-making quality, such as: decisional conflict, knowledge of treatment options and outcomes, perceived involvement in decision-making, and (hypothetical) uptake of evidence-based treatments which are congruent with patient preferences/values (i.e., informed choice). Importantly, the DA appears to be an appropriate resource for its target population, given that there were few differences between patients with a recent diagnosis (i.e., the target DA population) and those with a longer-standing diagnosis. Taken together, these findings are informative for the design of a future planned RCT to determine the DA’s potential efficacy at improving BPII treatment decision-making compared to usual care.

### Acceptability

Both quantitative and qualitative feedback confirmed that the DA had high acceptability amongst this sample of potential end-users. High acceptability is not surprising given that the DA’s content and format adhered to expert consensus-based international criteria (i.e., IPDAS) [22], were informed by the unmet informational and decision-support needs of potential end users [15], and were subject to rigorous iterative review by key stakeholder groups [59]. Moreover, strong endorsement of the DA among potential end-users is likely to support its successful future uptake and implementation in clinical settings, which is a challenge many decision-support interventions encounter [60].

Although participants uniformly endorsed the DA’s usefulness in treatment decision-making in general, some patients and family members indicated that the DA did not provide them with any new information nor facilitate treatment discussions with their family and/or treating clinician. A possible explanation of these findings is that the current high information-seeking, health literate sample had actively sought out and/or been provided with most of the DA-based information in the earlier stages of diagnosis, when this information is also most relevant. Further, this DA, like others [37], was designed to target a *specific* treatment decision at a *specific* point in the BPII trajectory. It was therefore beyond the DA’s scope to address other potential relation-based factors acting as supports or barriers to treatment decision-making, such as pre-existing family tensions and the strength of the therapeutic relationship [15, 35, 36], which are posited as especially important for shared decision-making (SDM) in mental health [61]. Although DAs are tools designed to facilitate SDM, they should not be considered synonymous with, nor sufficient for SDM [62]. Thus, embedding this DA in the broader care context may enhance its usefulness in supporting treatment discussions with clinicians. Indeed, about half of patients and family expressed a preference to use the DA in conjunction with their treating clinician. Clinicians are also likely to support using the DA in consultations, given that it incorporates a number of clinician-endorsed decision-support strategies [35], and its development involved substantial input from expert clinicians.

### Safety and feasibility

Participant feedback and self-report suggested that the DA content is not anxiety provoking and is therefore safe to use in this setting. State anxiety levels, although slightly elevated compared to non-clinical samples, were consistent with clinical norms for psychiatric samples [63], and were thus considered not specific to using the DA. Reinforcing this, the vast majority of patients and family indicated that reading the DA did *not* make them stressed or anxious. Those who did report experiencing some anxiety mostly attributed this to reading about adverse side-effects from medication. However, these participants, like other mental health patients [64], valued knowing this side-effect information and acknowledged that it was necessary for fully informed decision-making [46]. These findings align with those from a recently published Cochrane review of DA effectiveness, which indicate that exposure to a DA does not result in increased anxiety levels [37].

This pilot also demonstrated that the DA’s provision to these patients (and family) is feasible. Firstly, the chosen recruitment strategies resulted in a large proportion of patients with a recent BPII diagnosis who were currently considering or open to reconsidering their treatment options. These patients are at the decision-making stage whereby DAs are most useful [51] and thus form the DA’s target population. Secondly, response rates for both the patient (61.2%) and family (50%) samples were above the weighted average for similar research in counseling and clinical psychology, 49.6% [65]. Thirdly, both participant groups also indicated good engagement with the DA, with all indicating that they read the DA, with most reading it thoroughly. These encouraging response rates and high engagement with the DA suggest that the pilot procedure did not present any major barriers to patient/family participation, and provide preliminary support for the DA’s delivery within a community-based clinical setting.

### Potential usefulness

In addition to participant feedback, the DA’s potential usefulness was also supported by well-established measures of DA effectiveness [42]. After reading the DA, both patient and family were highly knowledgeable about treatment options and outcomes, based on current national guidelines on informed patient consent to medical interventions [46]. Namely, increased knowledge is one of the primary outcomes for assessing DA effectiveness [37], and has consistently been identified as enabling patient participation in decision-making and treatment uptake [66]. A majority of patients (65.5%) also made a decision that was congruent with their informed treatment values for medication, and half of patients (50%) for adjunctive psychological treatments, respectively). This said, the remaining patients made a treatment choice that was *not* consistent with their treatment attitudes. This finding was mainly attributable to patients being knowledgeable about treatment options, and choosing to take up medication with/out adjunctive psychological treatment despite their negative attitudes towards treatment. DAs are designed to target patient knowledge not attitudes. Therefore, this finding does not negate the value of this DA; i.e., helping patients to make informed, evidence-based choices. Indeed, greater knowledge of treatment side-effects and more realistic expectations of treatment benefits may indirectly impact on treatment attitudes. Furthermore, these informed choice rates compare favorably to RCT findings showing higher rates of informed choice amongst patients exposed to a DA for mammography (24%), [67], and bowel cancer screening (34%) [68], compared to usual care. Informed choice also represents an important DA outcome in the context of these ‘preference-sensitive’ decisions [37, 42].

In addition to making an informed choice, over 90% of patients made a treatment decision that was concordant with the best-available evidence (as per the DA). These high uptake rates closely align with those from a pre-/post- evaluation of an online DA for depression in young adults (93%) [69]. Of note too, similar proportion of patients chose to take up medication with/without adjunctive psychological treatment, which is encouraging as it provides support for the unbiased, non-directional nature of DAs [70], and patients’ awareness of choice [71]. These findings also challenge possible mental health clinician reluctance to engage patients in SDM, which stems from the concern that patients who receive balanced information on the adverse side-effects, and uncertain efficacy of available treatment options, would be less likely to accept evidence-based treatments [60, 72].

Paralleling these positive decision-making *outcomes*, the quality of the decision-making *process* was also high. After reading the DA, patients indicated feeling well-prepared to make treatment decisions and reported low levels of decisional conflict. This indicates that patients felt confident, well-informed and well-supported in decision-making, clear about their treatment values, and able to make an effective decision. Indeed, low decisional conflict has garnered amongst the most attention and support in the empirical literature on DA effectiveness [37], and is regarded as a hallmark attribute of decision-making quality [42]. Notably, the obtained decision conflict total and subscale means (< 25) are associated with patients more successfully following through with their treatment decision [44], which also aligns with one of the primary rationales for SDM, that SDM improves adherence to treatment [73]. These means also compare to those reported in RCTs where outpatients receiving a DA reported significantly lower decisional conflict for depression (*M* = 20.3) [9], (*M* = 23.85) [10] or PTSD treatments (*M* = 32.5), [12], compared to usual care. By contrast, the uncertainty subscale mean (> 25) indicated that some patients were feeling uncertain about their treatment decision after reading the DA. Other RCTs of mental health DAs report higher means or larger ranges on the uncertainty subscale relative to the other decisional conflict subscales [9, 10]. However, elevated levels of uncertainty are not necessarily unexpected or undesirable in this context, as they may reflect that the DA increased patient’s knowledge and thus afforded them better understanding of inherent uncertainty in the treatment options, and greater awareness of choice between numerous available options.

Another key outcome of DA effectiveness in decision-making is increased patient perceptions of involvement [37]. Consistent with this, only a small proportion of participants reported experiencing clinician-led decision-making in both dyadic and triadic consultations. However, it was not possible to determine whether patient and family reports of experienced involvement referred to consultations they attended before or after using the DA. That said, almost half of patients and two thirds of family member reported *not* experiencing their preferred level of patient involvement. This lack of concordance, either due to experiencing a more active or passive decision-making role than desired, may be especially pronounced in patients with bipolar disorder [24] who desire higher levels of involvement compared to other psychiatric patients but demonstrate fewer “active” behaviours (e.g., question-asking) in consultations [74]. Determining the DA’s effectiveness at improving the concordance between patients’ preferred and experienced involvement remains an important avenue for future intervention research. Indeed, concordance is associated with lower patient unmet needs, which in turn influence outcomes relevant to treatment adherence [54] such as the therapeutic relationship and quality-of-life [75].

Of note, pilot findings suggest the selected validated and purpose-designed measures were appropriate. Participants did not appear to encounter problems self-administering these measures (e.g., few missing data), and observed means/standard deviations aligned with similar DA RCTs [9, 10]. Other DA evaluation measures, such as satisfaction with decision and decisional regret [37], may serve as important additions to a future RCT to assess the DA’s longer-term impact on patient outcomes.

Finally, to evaluate the DA’s use in a future RCT using a larger, more representative patient sample, it is necessary to consider appropriate design changes to accommodate individuals who are more symptomatically-impaired, less health literate, and/or have fewer resources than the current pilot sample. Based on the PEMAT assessment [39], recommended changes to further strengthen the DA’s usability and understandability for individuals with low health literacy levels, (i.e., items scoring 0 or “disagree”) include: removing information or content that distracts from the DA’s purpose; using more common everyday language (e.g., replacing the following; pg. 18: “circumstances” with “life situation”; pg. 25 “minimise” with “reduce as much as possible”); and ensuring that all visual aids have clear titles and/or captions (e.g., adding titles and captions to all graphs and diagrams). For lower functioning individuals, the DA has the potential to be used during discussions with their clinicians and their families. Indeed, some patients and family (n = 16 and 1, respectively) indicated a preference for in-consultation use in the current pilot study.

### Limitations

Some limitations include the ‘opt-in’ recruitment methods, with the potential for self-selection bias. Secondly, the current findings may not generalise to patients and family with lower education, symptom-related functioning and/or health literacy levels. Nor may findings fully capture the preferences and decision-making characteristics of patients who are *actively* considering their treatment options, given that the majority of patients had already made a treatment decision by the time they reviewed the DA. Of note though, there were no apparent differences between participants who were symptomatic and those who were euthymic, nor between participants who were currently considering their treatment options and those who had made a treatment decision in the past. This lack of differences may be due to the fact that patients experiencing acute mood symptoms were excluded from the research, and that this self-selecting sample was likely more interested in/engaged with the treatment decision-making process regardless of whether or not they had already made a treatment decision. This said, as a pilot study, the small sample size (30 patients, 10 family members) precluded any formal statistical analyses of between-group differences.

Further, the current pilot design was not able to determine whether using the DA led to improvements on patient/family outcomes (e.g., high knowledge, low decisional conflict) because outcomes were assessed only at post-DA use and it did not include a control group. A future RCT phase will clarify any DA-specific improvements.

### Conclusion

This innovative DA addresses numerous unmet decisional-support needs identified by patients with BPII and their family [15], and adds to the relative paucity of evidence-based interventions for promoting SDM in mental health [76, 77]. Supporting the pilot aims, the DA was highly acceptable among potential end-users, and was feasible and safe to deliver to newly-diagnosed patients who are considering their treatment options to prevent relapse. Assessed factors related to both quality of the decision-making *process* (e.g., decisional conflict) and *outcomes* (e.g., knowledge and values-concordant choice) confirmed the DA’s potential usefulness for supporting informed treatment choices in the BPII setting.

## Supporting information

S1 Appendix DA content. (DOCX)

S2 Appendix Interview questions. (DOCX)

S3 Appendix Knowledge questions. (DOCX)
