# Supplementary material for: Development and pilot of a decision-aid for patients with bipolar II disorder and their families making decisions about treatment options to prevent relapse
Source: PLoS One. 2018 Jul 10;13(7):e0200490. doi: 10.1371/journal.pone.0200490 (PMC6039033; doi:10.1371/journal.pone.0200490)
Supplement: S1 Appendix — (DOCX) [file pone.0200490.s001.docx]

**Supplementary File 1.** Summary of decision-aid (DA) contents

| **DA section** | **Outline of included content** |
| --- | --- |
| **General introduction** | Outlined the purpose of DA, intended use/patient group, available treatment options |
| **Bipolar II disorder background** | Outlined types and prevalence of bipolar disorder, describing mood cycles, and types of treating clinicians |
| **Introduction to medication options** | Introduced the three first-line medication options (lithium, lamotrigine, quetiapine) in text and via flowchart, their effectiveness and ongoing, iterative nature of treatment decision-making |
| **Medication option 1: Lithium** | Introduced lithium, when lithium is recommended, its effectiveness at preventing different types of relapse via text and 100 person dot diagrams; outlined possible advantages (benefits) and disadvantages (side-effects/risks) of lithium over short, medium, long-term; included bona-fide patient/family member quotes relating to perceived pros and cons of lithium |
| **Medication option 2: Lamotrigine** | See above for lithium, with rewording as appropriate |
| **Medication option 3: Quetiapine** | See above for lithium, with rewording as appropriate |
| **Summary table of advantages/disadvantages of medication options** | Tabulated summary using traffic light info-graphic and colour-coding to denote advantages and disadvantages of lithium, lamotrigine, and quetiapine |
| **Introduction to add-on (adjunctive) psychological options** | Introduced the two level-1 evidence adjunctive psychological options (CBT and group psycho-education) in text and via flowchart, the rationale for having psychological treatment in addition to medication for relapse prevention, their key/overlapping components. |
| **Psychological option 1: Cognitive Behavioural Therapy (CBT)** | Introduced CBT, when CBT is recommended, its effectiveness at preventing different types of relapse via text and 100 person dot diagrams; outlined possible advantages (benefits) and disadvantages (side-effects/risks) of CBT; included bona-fide patient/family member quotes relating to perceived pros and cons of CBT |
| **Psychological option 2: Group Psycho-education** | See above for CBT, with rewording as appropriate |
| **Summary table of advantages and disadvantages of add-on psychological options** | Tabulated summary using traffic light info-graphic and colour-coding to denote advantages and disadvantages of CBT and group psycho-education |
| **What is the role of complementary therapy?** | Defined complementary therapy; introduced Omega-3 fatty acids and outlined current state of evidence in terms of relapse prevention |
| **How can family members be involved in decision-making?** | Outlined the potential roles and benefits of family involvement in decision-making both within and outside consultations with clinicians |
| **Making treatment decisions that are right for you** | Step-by-step guide on things to do/consider when making a treatment decision |
| **Making the most of your time with your clinician** | Gave examples of and addressed common patient barriers to asking clinicians questions; included “Ask-Share-Know” questions and question prompt list |
| **Worksheets: What is important to you about your treatment?** | Included values clarification exercises (with weight scale visual aids) for each medication and psychological treatment option, patient examples, and suggestions for involving family members and clinicians in completing these exercises |
| **Further resources** | Provided list of links to Australian-based websites/online resources |
| **Glossary of key terms** | Defined in lay language all medical/clinical terminology in the DA |
| **Acknowledgments** | Named and acknowledged development team, members of expert working party; indicated month/year that information is current, month/year of next planned update |
| **Reference list and further research** | Outlined the type of research/evidence used to base included treatment options and inform treatment efficacy data, list of key empirical studies/reviews |
