# Supplementary material for: Development and pilot of a decision-aid for patients with bipolar II disorder and their families making decisions about treatment options to prevent relapse
Source: PLoS One. 2018 Jul 10;13(7):e0200490. doi: 10.1371/journal.pone.0200490 (PMC6039033; doi:10.1371/journal.pone.0200490)
Supplement: S2 Appendix — (DOCX) [file pone.0200490.s002.docx]

**Appendix A.** Interview guide

**Question 1: Initial Response**

a) In your words what do you think is the purpose of this booklet?

b) What were your first impressions of this booklet?

c) What did you like about it? What was the best part?

d) What did you dislike about it? How can we improve on it?

e) Was there anything in the booklet that made you stressed or anxious?

f) Overall do you think a booklet like this is useful for a person to use when they are trying to decide about medication and psychological options to prevent relapse in bipolar II?

**Question 2: Design**

Do you have any comments about the ‘look’ or ‘design’ of the booklet, e.g., the colours; the images; the size of the writing; anything about the way it is presented?

**Question 3: General length and content**

a) What did you think about the length of the decision aid? Was it:

Too long

The right length

Too short

b) Was there:

Too much information

The right amount of information

Not enough information

**Question 4: Assistance with booklet**

Would it have been helpful if a clinician went through some of the pages with you before you looked through the booklet? If so, which ones?

**Question 5: Wording**

Were there any sentences or sections in the booklet that could have been clearer?

**Question 6: Now we will go over specific sections**

a) Turn to pages 16-17: Was the explanation of the different medication options clear?

b) On pages 42-43: Was the explanation of the different add-on psychological options clear?

c) Did you like that we included other people’s comments (in grey italics) on the pros and cons of each option?

d) Turn to pages 16 and 42: Are you clear about the options available?

Yes

No

e) In your own words, What are the options available (tick if mention option):

Medications (lithium, lamotrigine, quetiapine)

Adjunctive psychological treatments (CBT, group psycho-education)

f) What did you think of the 100 person dot diagrams (showing how effective options are at preventing relapse)? On pages 27 – 29: As an example, can you go through with me what each of these diagrams mean?

g) Do you have any comments about the description of the advantages/ benefits of each treatment option? How about in the summary tables?

h) Do you have any comments about the description of the disadvantages/ side-effects of each treatment option? How about in the summary tables on pages 40-41 and pages 60-61?

i) In terms of the presentation of the options, would you say that it favoured any particular option or did it provide a balanced view:

Taking a particular medication over others

(State which: ____________________)

Having a particular adjunctive psychological treatment over another

(State which: ____________________)

Balanced view

j) On pages 63 - 65, titled Information for family members: was this section useful?

k) Page 71: Worksheets:

i) Were the instructions on how to use the worksheets clear?

ii) Do you think the worksheets are a good idea? Did you find them useful?

l) Page 84: Further resources:

i) Would you access any of these websites?

ii) Would you recommend any other websites?

**Question 7 - Comprehensiveness**

a) Are there any topics or questions that you feel were not covered in the booklet that should be included?

Yes (i) If yes, please tell us what you think should be added)

No

b) Are there any topics or information that you think should not have been included in the booklet?

Yes (ii) If yes, please tell us what you think should be removed?)

No
