# Supplementary material for: Development and pilot of a decision-aid for patients with bipolar II disorder and their families making decisions about treatment options to prevent relapse
Source: PLoS One. 2018 Jul 10;13(7):e0200490. doi: 10.1371/journal.pone.0200490 (PMC6039033; doi:10.1371/journal.pone.0200490)
Supplement: S3 Appendix — (DOCX) [file pone.0200490.s003.docx]

Appendix B. Summary of purpose-designed knowledge items and scoring

| **NHMRC guidelines on information to be given for informed consent (1)** | **DA-related Conceptual (gist) knowledge – 9 questions x 2 marks (/18)** | **DA-related Numerical (verbatim) knowledge – 5 questions x 4 marks (/20)**  **“Adequate knowledge”** is defined as a pass mark of > 50% (i.e., at least 20 out of 38 marks)  To have adequate knowledge, participants must either:   - Get correct all conceptual/gist knowledge items (18 marks) ***plus*** at least 2 marks on numerical/verbatim knowledge items (2 marks)   **ALTERNATIVELY**   - Get correct all numerical/verbatim knowledge items (20 marks).   In this way, participants cannot have “adequate knowledge” on the basis of conceptual/gist knowledge alone, instead they either need to have a combination of conceptual/gist and numerical/verbatim knowledge, or numerical/verbatim knowledge alone (2, 3).  Note that each applicable NHMRC guideline was assessed with a conceptual and/or numerical question. |
| --- | --- | --- |
| **1) The possible of likely nature of the illness or disease** |  | **1)** Over the long term, *how much of the time will the average person with BPII spend WITHOUT ANY SYMPTOMS*?  ANSWER: 24 weeks per year (46% of the time) without any symptoms.  Almost one half of the time (~ 24 weeks per year) = 4 marks  Just over one third of the time (~ 19 weeks per year) = 3 marks  Less than one fifth of the time (~ 8 weeks per year) = 2 marks  Almost never (1 - 2 weeks per year) = 1 mark  Almost all the time (45 -50 weeks per year) = 0 marks |
| **2) The proposed approach to treatment:**   1. **what the proposed approach entails** 2. **the expected benefits** 3. **common side effects and material risks of any intervention** 4. **whether the intervention is conventional or experimental** 5. **who will undertake the intervention** | **2) i)** Taking medication *together with* psychological treatments is more effective than medication *only* for preventing relapse in BPII.  True (correct) = 2 marks  False/Don’t know = 0 marks  **2) ii)** Lithium, lamotrigine, and quetiapine differ in terms of how effective they are at preventing hypomania.  True (correct) = 2 marks  False/Don’t know = 0 marks  **2) iii)** Lamotrigine is associated with sedation, weight gain, and sleepiness/drowsiness.  True/Don’t know = 0 marks  False (correct) = 2 marks  **2) iv)** N/A  **2) v)** In Australia, clinical psychologists have training to provide cognitive behavioural therapy (CBT).  True (correct) = 2 marks  False/Don’t know = 0 marks | **2) ii)** Imagine a group of 100 people taking quetiapine. *About how many people taking quetiapine* will *RELAPSE in general*?  ANSWER: 23 in 100 people will experience relapse in general.  About one quarter (~25 in 100 people) = 4 marks  About one third (30-40 in 100 people) = 3 marks  Almost one half (45-50 in 100 people) = 2 marks  Less than fifth (10-15 in 100 people) = 1 marks  Almost everyone (~90 in 100 people) = 0 marks  **2) iii)** Imagine a group of 100 people starting lamotrigine.  *About how many in 100 people* will experience non-serious (benign) rash within the first 2 months?  ANSWER: 8-9 in 100 people  Almost one tenth (5-10 in 100 people) = 4 marks  About one quarter (~25 in 100 people) = 3 marks  About one third (30-40 in 100 people) = 2 marks  About one half (~50 in 100 people) = 1 mark  More than three quarters (75+ in 100 people) = 0 marks  **2) iv)** N/A |
| **3) Other options for treatment** | **3)** Lithium, lamotrigine, and quetiapine are the only available medication options that your clinician will recommend to you.  True/Don’t know = 0 marks  False (correct) = 2 marks |  |
| **4) The degree of uncertainty of any diagnosis arrived at** | N/A (not related to diagnosis) | N/A (not related to diagnosis) |
| **5) The degree of uncertainty about the therapeutic outcome** | **5)** We still do NOT know which out of lithium, lamotrigine, and quetiapine is *the most* effective at preventing relapse in BPII.  True (correct) = 2 marks.  False/Don’t know = 0 marks. |  |
| **6) The likely consequences of not choosing the proposed treatment, or of not having any treatment at all.** | **6)** A person with BPII who does *NOT* take any medication is at greater risk of relapse compared to a person with BPII who *DOES* take medication.  True (correct) = 2 marks.  False/Don’t know = 0 marks. | **6)** Imagine a group of 100 people treated with medication but WITHOUT any add-on or adjunctive psychological treatment.  *About* *how many in 100 people* will relapse within 2 years?  ANSWER: 50 in 100  About half (~50 in 100 people) (i.e., 50 > 10%)= 4 marks  About one third (30-40 in 100 people) (i.e., 50 +/- 25%) = 3 marks  About one quarter (~25 in 100 people) (i.e., 50 +/- 50%) = 2 marks  More than three quarters (~80 in 100 people) (i.e., 50 +/ - 75%) = 1 mark.  Almost everyone (~99 in 100 people) (i.e., 50 +/- 100% +) = 0 marks. |
| **7) Any significant long term physical, emotional, mental, social, sexual or other outcome that may be associated with a proposed intervention.** |  | **7)** Imagine a group of 1000 people taking lithium over the long-term.  *How common* is it for these people to experience complete kidney (renal) failure?  ANSWER: 5 in 1000  Uncommon (1-9 in 1000 people) = 4 marks  Rare (Less than 1 in 1000 people) = 3 marks  Common (50-100 in 1000 people) = 2 marks  Very common (~500 in 1000 people) = 1 mark  Experienced by almost everyone (~900-990 in 1000 people) = 0 marks. |
| **8) The time involved.** | **8)** The actual number of sessions in psychological therapies (cognitive behaviour therapy and group psycho-education) varies from one person to another or from one group to another.  True (correct) = 2 marks.  False/Don’t know = 0 marks. |  |
| **9) The costs involved, including out of pocket expenses.** | **9)** Lithium, lamotrigine, and quetiapine are *all* subsidised by the PBS, and so cost a similar amount.  True/Don’t know = 0 marks.  False (correct) = 2 marks. |  |

1. Australian Government, National Health and Medical Research Council. General guidelines for medical practitioners on providing information to patients2004.

2. Smith SK, Barratt A, Trevena L, Simpson JM, Jansen J, McCaffery KJ. A theoretical framework for measuring knowledge in screening decision aid trials. Patient education and counseling. 2012;89(2):330-6.

3. Smith SK, Trevena L, Simpson JM, Barratt A, Nutbeam D, McCaffery KJ. A decision aid to support informed choices about bowel cancer screening among adults with low education: randomised controlled trial. BMJ. 2010;341:c5370.
